# Phonological discrimination and contrast detection in pupillometry

**DOI:** 10.3389/fpsyg.2023.1232262

**Published:** 2023-11-01

**Authors:** Julia S. C. Chiossi, François Patou, Elaine Hoi Ning Ng, Kathleen F. Faulkner, Björn Lyxell

**Affiliations:** ^1^Oticon A/S, Smørum, Denmark; ^2^Department of Special Needs Education, University of Oslo, Oslo, Norway; ^3^Oticon Medical, Smørum, Denmark; ^4^Department of Behavioural Sciences and Learning, Linnaeus Centre HEAD, Swedish Institute for Disability Research, Linköping University, Linköping, Sweden

**Keywords:** pupillometry, speech perception, phoneme perception, acoustic cues, novelty detection, linguistic context

## Abstract

**Introduction:**

The perception of phonemes is guided by both low-level acoustic cues and high-level linguistic context. However, differentiating between these two types of processing can be challenging. In this study, we explore the utility of pupillometry as a tool to investigate both low- and high-level processing of phonological stimuli, with a particular focus on its ability to capture novelty detection and cognitive processing during speech perception.

**Methods:**

Pupillometric traces were recorded from a sample of 22 Danish-speaking adults, with self-reported normal hearing, while performing two phonological-contrast perception tasks: a nonword discrimination task, which included minimal-pair combinations specific to the Danish language, and a nonword detection task involving the detection of phonologically modified words within sentences. The study explored the perception of contrasts in both unprocessed speech and degraded speech input, processed with a vocoder.

**Results:**

No difference in peak pupil dilation was observed when the contrast occurred between two isolated nonwords in the nonword discrimination task. For unprocessed speech, higher peak pupil dilations were measured when phonologically modified words were detected within a sentence compared to sentences without the nonwords. For vocoded speech, higher peak pupil dilation was observed for sentence stimuli, but not for the isolated nonwords, although performance decreased similarly for both tasks.

**Conclusion:**

Our findings demonstrate the complexity of pupil dynamics in the presence of acoustic and phonological manipulation. Pupil responses seemed to reflect higher-level cognitive and lexical processing related to phonological perception rather than low-level perception of acoustic cues. However, the incorporation of multiple talkers in the stimuli, coupled with the relatively low task complexity, may have affected the pupil dilation.

## 1. Introduction

The perception of contrast between phonemes is a fundamental aspect of speech perception and the basis for language acquisition (Kuhl et al., 2008; Casserly and Pisoni, 2010). By gradually extracting patterns from speech, infants learn to divide acoustic input into phonetic categories and sequences of phoneme combinations into words (Kuhl et al., 2008; Romberg and Saffran, 2010). As vocabulary grows, consistent perception of acoustic/phonetic patterns in the speech input will activate lexical processing for word recognition or pathways for learning a novel word (Pittman et al., 2017). However, the perception of phonological contrasts is not uniquely driven by its acoustic properties. Phonological perception evolves to accommodate predictions from the linguistic context and the inherent phonetic variability in speech (Repp, 1982; Allen et al., 2003; Clarke and Garrett, 2004; Jesse, 2021). In terms of cognitive processing, low-level acoustic perception and high-level lexical processing are integrated to determine the presence or significance of a specific contrast (Borsky et al., 1998; Coleman, 2003).

Low-level processing involves interpreting the acoustic properties of speech sounds. Acoustic cues refer to distinct auditory features that convey information. Phoneme contrasts are marked by a variety of cues in spectral and temporal acoustic dimensions. These acoustic cues are redundant, such that several distinct cues occur for a particular contrast and can be traded with each other for the perception of a particular phoneme (Liberman et al., 1967; Repp, 1982; Winn et al., 2012). As an example, place of articulation for stop consonants can be cued by F2 and F3 transitions, burst frequency, or burst amplitude (Repp, 1982, for multiple examples). These cues covary in natural speech, and listeners must integrate them to achieve the most reliable identification of the incoming speech stimuli. Although this process may remain robust while a cue is missing or degraded, it would be expected that the demand for cognitive processing would increase with higher ambiguity of the stimulus and degradation of differential cues.

High-level phonological processing is guided by top-down knowledge of linguistic rules and context (Casserly and Pisoni, 2010; Jesse, 2021). The presence of high-level processing on phoneme discrimination leads to a perceptual bias in which listeners disambiguate underspecified phonemes toward meaningful compositions (Ganong, 1980; Jesse, 2021). For example, individuals may identify ambiguous speech sounds as a real word rather than a nonsense word if context is present.

For this high-level component in phonological perception, it is challenging to separate the specific contribution of each type of processing on task accuracy, as both high-level and low-level processing may be involved in successful performance. However, an ambiguous stimulus, which theoretically requires higher levels of processing, would likely demand the allocation of more cognitive resources, thus increasing listening effort (Kramer et al., 2013; Johnsrude and Rodd, 2016). Therefore, having an objective method that is sensitive to the individual demands for both low and high-level phonological processing could help to explain the variability in speech perception performance attributed to hearing impairment and challenging auditory environments (Phatak and Grant, 2014; Gianakas and Winn, 2019).

Assessing cognitive processes objectively during phonological contrast perception involves measuring responses to speech stimuli occurring at a cortical level. Physiological markers like late-latency auditory event-related potentials and mismatch negativity (MMN) have provided evidence of a pre-attentive component of phonological discrimination which could capture changes in the phonological pattern before conscious perception (Näätänen et al., 2007; Steinhauer and Connolly, 2008). However, cortical measures can be time-consuming and uncomfortable for participants. As an alternative, pupillometry could be used as a tool to investigate the temporal dynamics between low- and high-level processing of phonological stimuli. The pupil dilation is linked to increasing the norepinephrine release from the locus coeruleus, an area associated with attentional prioritization and perception of novelties (Eckstein et al., 2017; Kafkas and Montaldi, 2018). Task-evoked pupil dilation is measured by videorecording the pupil size during a task. It has the advantages of being cost-effective and providing good temporal resolution (Winn et al., 2018). In terms of cognitive processing, at a low level, pupil dilation is sensitive to the perception of novelty that arises from a mismatch between stimulus and context, as changes in the stimulus frequency, intensity or pitch (Virtala et al., 2018; Bala et al., 2020). At a high level, pupil dilation has been shown to increase with linguistic processing demand, working memory load, and the effort required to resolve ambiguity in speech (Wendt et al., 2016; Zekveld et al., 2018; Kadem et al., 2020; Micula et al., 2022).

Previous research on the use of pupillometry to assess the discrimination of speech sounds, particularly phonemes, is limited and mainly focused on perception within word or sentence context (Wagner et al., 2016; Kinzuka et al., 2020; Winn and Teece, 2021). Kinzuka et al. (2020) used an oddball paradigm to evaluate the correlation between the perceptual ability of Japanese speakers to discriminate the /r/ and /l/ sounds in real words and their pupillometric responses. The study found that higher language proficiency is associated with earlier occurring differences in peak of pupil dilation (PPD) between target frequent and infrequent stimuli. In another study addressing the effects of phonological manipulation on pupil dynamics, Winn and Teece (2021, 2022) measured pupil dilation during sentence recognition using embedded phonologically altered words in both normal hearing and cochlear implant subjects. The authors reported a steeper increase in pupil size in response to phonological alterations, with larger differences when the target phoneme was substituted by noise instead of another phoneme. For cochlear implant users, the contrast in pupil responses between sentences with and without phonological substitutions was shallower than for normal hearing peers, suggesting a relationship between degraded speech perception and the pupil response. This effect of speech degradation was also observed by Wagner et al. (2016), who reported steeper pupil dilation curve slopes for unexpected word prosody in full-frequency-spectrum speech but not for cochlear implant simulated speech.

It is important to note that in the paradigms described above, participants’ attention was directed toward processing the entire sentence or word, actively engaging high-level processing to interpret its meaning. However, the presence of context makes it difficult to distinguish the pupil variation caused by the perception of a phonological contrast, from that caused by the perception of lexical meaning variation which is known to cause pupil dilation independently (Kamp and Donchin, 2015). Additionally, sentence comprehension in adverse listening conditions is known to produce higher pupil dilation (Wendt et al., 2016; Ohlenforst et al., 2018; Trau-Margalit et al., 2023). Therefore, directing attention to the whole sentence might obscure the responses related to phonological contrast detection, since pupillometry seems to reflect responses to attended stimuli rather than passive listening (Kramer et al., 2013) and is larger for perceived rather than unperceived errors (Kamp and Donchin, 2015). On the contrary, it is possible that by directing attention to the presence of phonologically altered stimuli, responses may better reflect the low-level processing independently of the presence of context.

This study aimed to investigate the pupil temporal dynamics during the auditory processing of phonological contrasts, in an effort to differentiate low- and high-level processing of phonological information. For that, two paradigms were contrasted. First, we investigated the pupillometric response to low-level processing of phonological contrasts, measured during the perception of lexically decontextualized phoneme contrasts (phonological discrimination task). Second, we explored the possibility to record similar responses in the presence of lexical information, without prompting high-level sentence processing (detection task). Additionally, to introduce an acoustic challenge to the perception of the phonological contrast, we investigate how those responses are impacted a sub-optimal speech input, using a vocoded speech signal.

To minimize the influence of lexical knowledge on phonological perception, we chose to explore phonological contrasts using nonwords as our tokens. This approach aimed to preserve the real-world relevance of word-like items, while removing their lexical meaning. Traditional methods for assessing phonological identification and recognition often employ syllabic continua (Iverson, 2003; Abada et al., 2008; Lewis and Bidelman, 2020). However, syllables may not demand the same level of cognitive processing for their phonological contrasts as longer stimuli. Therefore, we chose to use nonwords, as they would enhance the ecological validity of the pupillometry measures.

We hypothesized that if pupil dynamics were sensitive to the low-level acoustic properties of a phonological contrast, larger pupil dilations would be measured in conditions where the phonological contrast is present. These differences would be maintained independently of the presence of context and under a vocoded speech signal, whenever the phonological contrast was correctly perceived. However, if the pupil dynamics reflect the high-level linguistic processing required to disambiguate a phonological contrast, larger pupil dilations would be measured for a phonological contrast only in the presence of lexical information. We expect that the results provided here enhance the understanding in pupillary responses to phonological processing, shedding light on their sensitivity to different processing levels and adverse speech conditions.

## 2. Materials and methods

### 2.1. Participants

A convenient sample was recruited across the researcher’s place of employment, in the Capital Region of Denmark. A sample of 22 adults (age: [25; 0–65;0], median: 50 years; females: 39%; all with more than 11 years of education) who reported Danish as their first language, and self-reported normal hearing, were included. Participation was voluntary, and the researchers were contacted directly by the participants after an online announcement in an internal website. Participants joined during working hours and were compensated with their regular salary for their time.

This study was waived from ethical review by the Regional Committee of Health Research Ethics - Capital Region, Denmark, after inquiry submission, as it was considered to be research in the social domain. All participants gave active consent to the study, after receiving written and oral information, in accordance with the Declaration of Helsinki. Requirements regarding the General Data Protection Regulation (GDPR) were carefully followed.

### 2.2. Stimuli

#### 2.2.1. Phonological discrimination task

The discrimination task was composed of two lists containing 40 pairs of disyllabic-nonwords, selected from the nonword corpus published by Nielsen and Dau (2019). From the original material, C−/a/-C−/a/ nonwords starting with one of the 14 main initial phonemes for the Danish language (/p t k b d g m n l f v s r h /) were selected. The recordings selected had 100% speech intelligibility score reported in the original study by Nielsen and Dau (2019), for both first and second consonants. The present study focuses only on the first phoneme contrast.

For the task, nonwords were combined in pairs, to account for all the minimal-pair combinations in Danish for which just one distinctive production feature is present (place, voice/aspiration, or manner, as in ‘*bafi – ‘pafi’*). To facilitate phonological ─ instead of acoustical ─ comparison of the word pairs, recordings of each nonword from three different speakers were selected from the original material and each pair was presented using audio from two different speakers, selected randomly among the three possible recordings. Post-hoc analysis revealed no effect of the speaker-pair used on participants’ performance.

All the audio recordings were normalized by root mean square (RMS) and silence was added in the beginning of each file to align the nonwords’ offset during the task and to randomize the nonword start and the interval between nonwords.

#### 2.2.2. Detection task

A second task explored the effect of context, in which the participants were asked to track a phoneme substitution in a word within a sentence (Pittman and Schuett, 2013). The detection task was composed of two lists of 36 four-word-sentences. The original sentences in the lists were composed of simple words from a 3 year-old child’s vocabulary (Bleses et al., 2008a, 2008b) to guarantee that the words included would be well known by the participants. The sentences were evaluated as highly meaningful by a group of 25 native Danish speakers, in a pre-study conducted by our group. For half of the sentences in each list, the first phoneme of the second word was substituted for another phoneme with similar phonotactic probability (e.g., *Hunden finder altid maden* [the dog always finds the food] - > *Hunden sinder altid maden*, an equivalent example in English would be ‘Dad buys new shirts’ - > ‘Dad fuys new shirts’, from Pittman and Schuett (2013)). We were careful to choose phonemes that would generate a nonword when replacing the original phoneme, which was confirmed by a group of 14 native speakers who listened to the generated nonwords in isolation and were asked to write the first real word it would remind them of (less than 50% of the participants could point to same original or other real word). Due to this requirement, the phonemes selected had contrasts in one or more production features with the original phoneme, which potentially added cues that may have aided detection in the sentence context. Moreover, to avoid that the second word in the sentence could be predicted by the sentence context, the same group of 25 native speakers were asked to complete the sentences where the target word was missing. Only the sentences with less than 10% of participants filling in the same real word (defined as low cloze probability in Kutas and Hillyard, 1984) were included in the lists.

The final 72 sentences, half with embedded nonwords, were recorded by a female native Danish speaker with an accent from the Danish capital region. She was instructed to pronounce the sentences in a natural prosody but in a slow speaking pace. All the recordings were normalized by RMS and silence was added in the beginning of each file to randomize the sentences’ start.

#### 2.2.3. Stimuli vocoding

In order to reduce the acoustic features, challenging the detection and discrimination of phonological contrasts (Stenfelt and Rönnberg, 2009), one list was randomly vocoded for each participant. The vocoding process includes dividing the speech signal into frequency bands, extracting the amplitude envelope for each band, and using it to modulate a noise band, resynthesizing the bands to create a new audio file. For this study, the vocoded versions of the stimuli were generated using the software Praat (Boersma and Weenink, 1992) and the open-source code provided by Winn (2021) (version 45). An 8-channel vocoder was used, with flat-spectrum noise-carrier, and corner frequencies set between 0.2-8 kHz. This number of bands was chosen to add challenges to the transmission of spectral information while approaching the asymptotic speech-recognition performance in quiet (Dorman et al., 1997; Friesen et al., 2001; Xu et al., 2005).

#### 2.2.4. Vocoded real-word recognition

Considering that a participant’s inability to recognize real words in the vocoded condition could influence their performance on nonword detection, vocoded word recognition scores were also calculated. The first list of the clinical test Dantale I (Elberling et al., 1989) in silence was vocoded using the method described above. Participants were presented with the recorded monosyllabic words in isolation and were asked to repeat them aloud. The participant’s response was recorded and transcribed, for offline scoring.

### 2.3. Pupillometry

Pupil size was continually measured by the Pupil Core® platform (Pupil Labs GmbH, Berlin). The glasses-mounted solution includes one front camera recording the gaze direction and two infra-red cameras that record the pupils at a sampling frequency of 200 Hz. Pupil tracking is done in dark mode. The software provides the pupil size for each eye in arbitrary units (pixels) and a confidence score, defined as an index, between 0 and 1, indicating the quality of the acquired value.

### 2.4. Procedure

The study protocol was implemented via computer on the OpenSesame platform (Mathôt et al., 2012), using the features developed by Sulas et al. (2022).

The experiment was conducted in an acoustically treated sound studio. Participants indicated their responses using a touchscreen monitor placed on a table in front of them. The monitor was positioned to have the top ¾ of the screen aligned to the participant’s eye. Sound was presented from a loudspeaker positioned 1 m directly in front of the participant (0-degrees Azimuth). Test participants wore the pupillometry glasses with the cameras adjusted so that the pupils were in the middle of the cameras respective field of view. The glasses were worn during the whole session and adjusted as needed between tasks in case of displacement. Lighting conditions and the screen luminance were kept constant at 200 lumens.

Prior to starting the experimental tasks, the participants were familiarized with vocoded speech. Sentences were presented back-to-back in non-vocoded and vocoded conditions, for about 3 min, until the participant reported feeling comfortable recognizing the sentence in the vocoded version. The full testing session included other speech perception tasks not reported here and took approximately 1.5 h. Task order and sequence were randomized to counterbalance fatigue effects. All tests were preceded by verbal and written instructions, plus a training phase during which direct verbal feedback and clarifications were provided.

**Figure 1:**
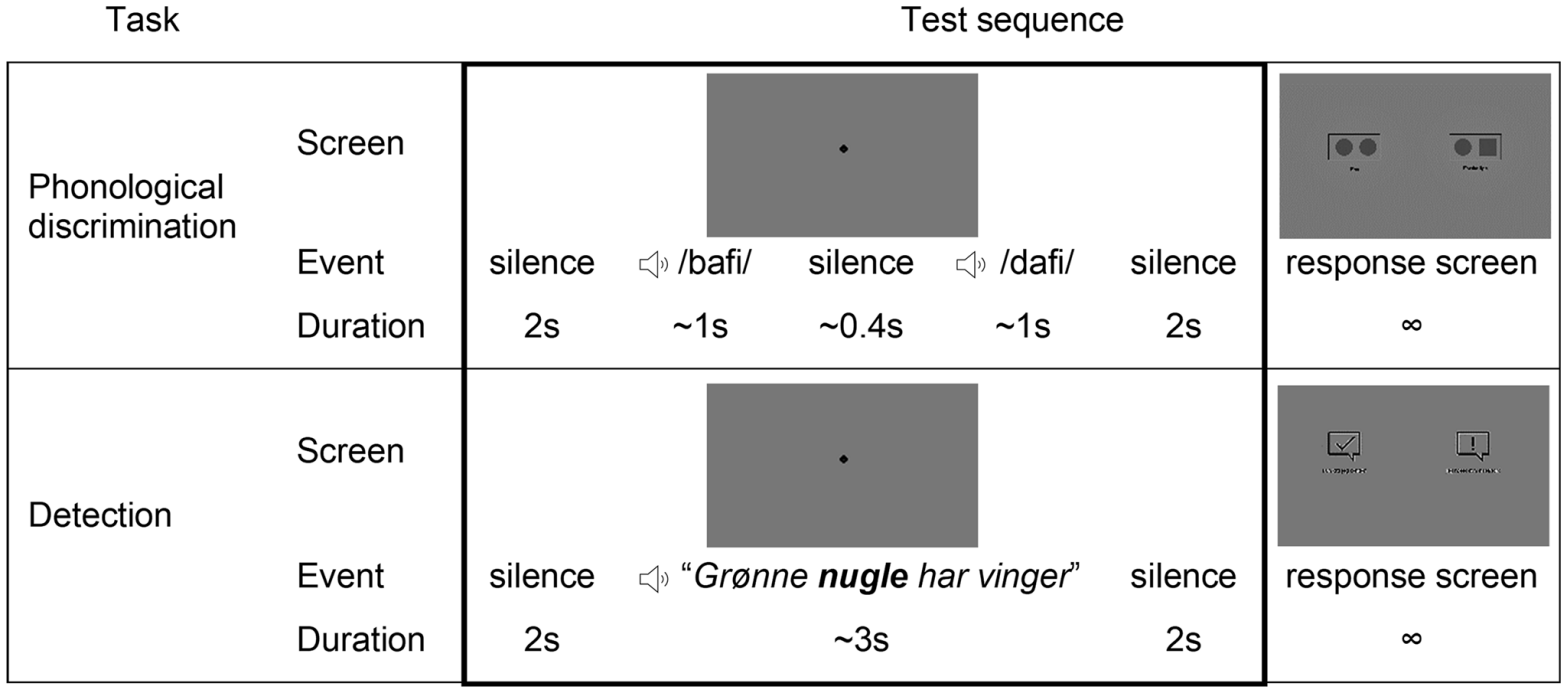
Example of sequence of screens and actions on the phonological discrimination and detection tasks.

In the phonological discrimination task, word pairs were presented one by one. The participant was asked to indicate if the second word in the pair was the same as the first in a ‘yes/no’ paradigm. A fixation dot was kept in the screen from 2 s before until 2 s after the presentation of each word pair (detailed in Figure 1). Participants were asked to look at the dot in order to reduce eye movements and improve the quality of the pupillometry data. After each pair presentation, participants indicated their response via touchscreen. This task took approximately 7 min to complete in each condition, and conditions were randomized across participants.

In the detection task, participants were asked to indicate if the sentence contained a nonword (the phonologically modified word) in a ‘yes/no’ paradigm. Participants listened to a list of 36 sentences in each condition. The trial sequence is illustrated in Figure 1. Two seconds of silence were added before and after each sentence, while a fixation dot was kept on the screen. Testing took approximately 5 min in each condition, and conditions were randomized across participants.

### 2.5. Analysis

#### 2.5.1. Task performance

For both phonological discrimination and the detection tasks, accuracy for ‘yes/no’ responses was recorded. Analysis was conducted in terms of signal detection theory (Macmillan and Creelman, 2005). The ‘signal’ in the stimulus was defined as the presence of a phonological contrast, namely, the presence of a nonword in the sentence or a phonological substitution in the second token of the nonword-pair. Responses were classified as ‘hits’: correct responses when the signal was present, ‘misses’: incorrect responses when the signal was present, ‘correct rejections’: correct responses when the signal was absent, and ‘false alarms’: incorrectly reporting the presence of the signal when it was absent. The proportion of correct responses was calculated as the sum of ‘hits’ and ‘correct rejections’, divided by the total number of trials (Macmillan and Creelman, 2005).

The discrimination score (*d’*) was calculated as measure of the participants’ sensitivity to the presence of a signal (Macmillan and Creelman, 2005). It was estimated by subtracting the *z*-transformed ‘hit’ rates and ‘false-alarm’ rates. To avoid floor and ceiling effects in *d’* calculation, a correction for the extreme values was performed using the log linear approach described by Stanislaw and Todorov (1999), by adding 0.5 to both the number of ‘hits’ and ‘false alarms’ and adding 1 to the number of trials, before calculating the *d’* score. Additionally, to analyze a possible response bias toward selecting one of the two options (‘yes’/‘no’), the criterion location was calculated as minus half of the sum of *z-*transformed ‘hit’ and ‘false-alarms’. A positive criterion value indicates a bias to ‘miss’ the signal although it is present, while negative values represent bias toward accusing the presence of the signal despite its absence (‘false alarms’). Together, *d’* and criterion location give a parameter of participants’ strategy in the phonological discrimination and detection tasks.

#### 2.5.2. Pupillometry pre-processing and analysis.

Pupil data were segmented by trial, and data were analyzed from the eye with best overall confidence during the task, calculated by the percentage of data points over 0.85 of confidence, as reported by the equipment software. The data were cleaned of blinks and artifacts by detecting dilation speed outliers with the method described by Kret and Sjak-Shie (2019) and excluding the flagged data points with a backward and forward margin of 50 ms. Data reconstruction was done using Piecewise Cubic Hermite Interpolating Polynomial (Pchip) or linear interpolation when the Pchip was not possible (where there were not enough points available before or after the region to interpolate), considered the good reconstruction properties of both methods reported by Dan et al. (2020). Blinks above 500 ms were not reconstructed. The individual data points were downsampled to 30 Hz, as the pupil response latency of is over 200 ms (Winn et al., 2018; Mathôt and Vilotijević, 2022), and smoothed using a moving-average filter of 0.1 s.

Trials with more than 45% interpolated data were excluded from the analysis (Burg et al., 2021; Zhang et al., 2022). Baseline pupil size was calculated per-trial by taking the mean pupil size during the 500 ms right before stimulus onset (Seropian et al., 2022). All subsequent data points in the trial were calculated as the proportional change relative to that baseline pupil size. As a last step, raw and processed data were visually inspected to identify and exclude trials with potential contamination, as artifacts in the baseline estimation period or absolute changes in pupil size over 40% of the baseline (Winn, 2016; Winn et al., 2018).

Subjects with more than 50% of the trials excluded from one task condition, had their results excluded from the analysis in that specific task. This criterion excluded pupillometric data from three subjects in both conditions of the phonological discrimination task only. For the remaining participants and tests, the aggregated trace of the pupil response for correct answers was calculated. Data was extracted regarding the value and time of the maximum pupil size – respectively, peak pupil dilation (PPD) and the peak pupil dilation latency (PPL) – from the time window spamming from the target stimulus onset (the second word) to 1 s after the audio offset. To compare with studies with similar methodology (Wagner et al., 2016; Winn and Teece, 2021), a growth curve analysis (GCA) was carried out, which models the quadratic fit of the pupil curve between the target-stimulus onset and the PPD.

#### 2.5.3. Inferential analysis

Inferential analysis was conducted in Python 3.9, using ‘SciPy’ (v. 1.7.3) and ‘Statsmodels’ (v. 0.13.2) packages. Normality in distribution was assessed using Shapiro–Wilk, for the subsequent choice of parametric or nonparametric statistical tests described in the results. Paired comparisons were conducted for mean/median comparison of signal detection performance (*d’*) in vocoded and non-vocoded conditions, and the sequential points in the pupillometric curve in ‘yes’ versus ‘no’ tasks. Logistic regression was used to investigate how GCA parameters (intercept, slope, and quadratic term) could be modeled to determine the type of pair (‘yes’ or ‘no’) identified. Additionally, effects of vocoding in the pupillometry metrics were analyzed using a linear mixed effect model in a matrix of auditory condition (‘vocoded’ or ‘non-vocoded’) and pair type (‘yes’ or ‘no’), with participants attributed as random effects. The inclusion of pair type in the model derives from the assumption that the detection of a phonological contrast in the target word (‘yes’ tasks) would produce a more prominent response in task evoked pupillometry (Kinzuka et al., 2020).

## 3. Results

### 3.1. Performance results

**Figure 2:**
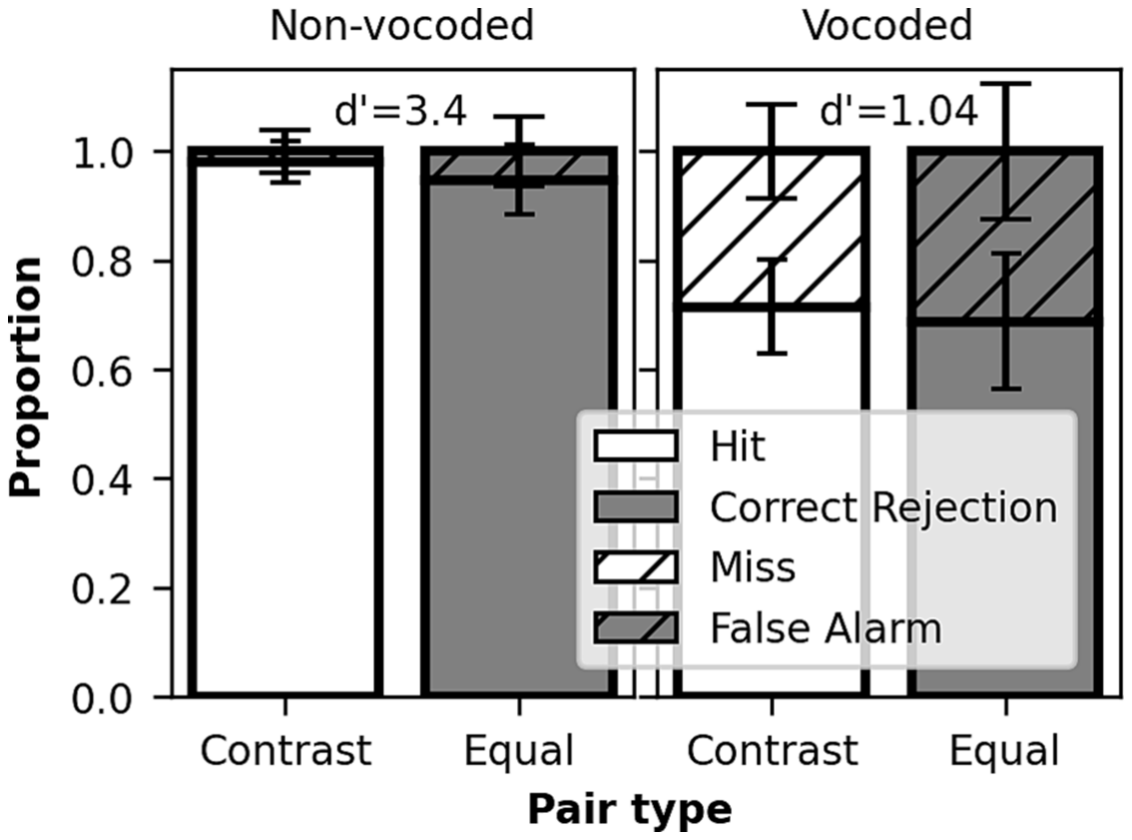
Performance in the discrimination task on vocoded and non-vocoded conditions. Stacked bar plot. White bars represent nonword pairs containing a phonological contrast in the second nonword and gray bars pairs with the same nonword. Full bars represent correct responses, while dashed bars represent errors.

**Figure 3:**
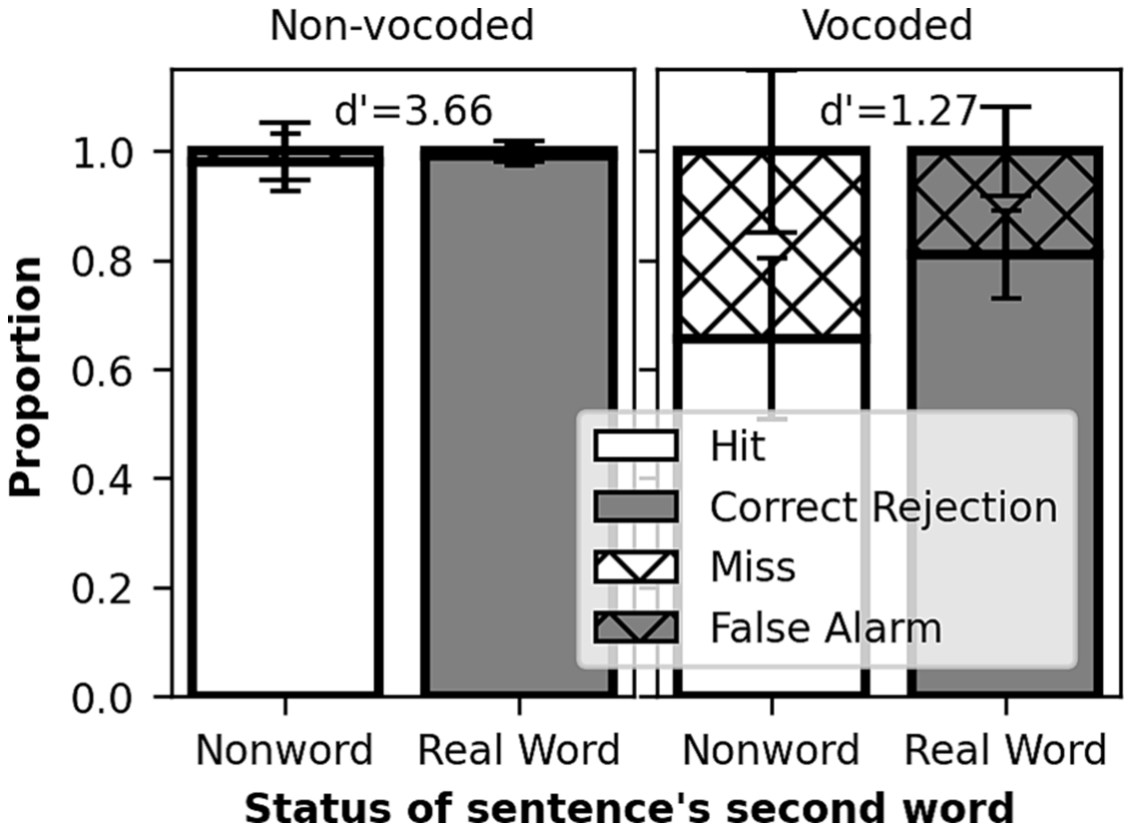
Performance in the detection task on vocoded and non-vocoded conditions. Stacked bar plot. White bars represent sentences containing a nonword and gray bars sentences without a nonword. Full bars represent correct responses, while dashed bars represent errors.

The participants had near ceiling scores on the perception of phonological contrasts for non-vocoded speech, with mean *d’* scores of 3.39 (SD = 0.56) for the phonological discrimination (Figure 2) and 3.66 (SD = 0.46) for the detection task (Figure 3). For vocoded speech, the performance decreased significantly in both tests, with mean d’ scores of 1.04 (SD = 0.48) for the phonological discrimination and 1.26 (SD = 0.54) for the detection task. The difference between non-vocoded and vocoded conditions was confirmed by paired comparison *t*-tests, *t* (21) = 16.15 for phonological discrimination and *t* (21) = 15.39 for detection task, *p* < 0.001 for both tasks. Despite lower scores, mean performance was above the 50% chance level in the vocoded condition (mean 70% correct responses for phonological discrimination and 73% correct responses for detection task), confirming that the participants were able to perform both tasks with the vocoded stimuli. Vocoded real-word recognition in the Dantale test had an average accuracy of 34% (SD = 16%). In a simple regression model, the word recognition of vocoded speech alone accounted for over 20% of the variance in the phonological discrimination *d’* scores, *R^2^* = 0.21, *F* (1,19) = 4.97, *p* = 0.04, but did not explain the variance in the detection task, *R^2^* = 0.01, *F* (1,19) = 0.14, *p* = 0.71.

Analyzing the effect of lexical context in the detection of a phonological contrast, Wilcoxon signed-ranks test showed no difference in performance with or without lexical context, when comparing the *d’* scores of the phonological discrimination and detection tasks, *z* = 80.0, *p* = 0.13. Nevertheless, in the response bias analysis, criterion was located positively at a mean of 0.24 (SD = 0.26) for the detection task, suggesting that participants were biased toward not detecting the nonword despite its presence, while for the phonological discrimination task, criterion was placed much closer to zero, at a mean of −0.04 (SD = 0.18), suggesting no bias on the response.

### 3.2. Pupillometry responses

**Figure 4:**
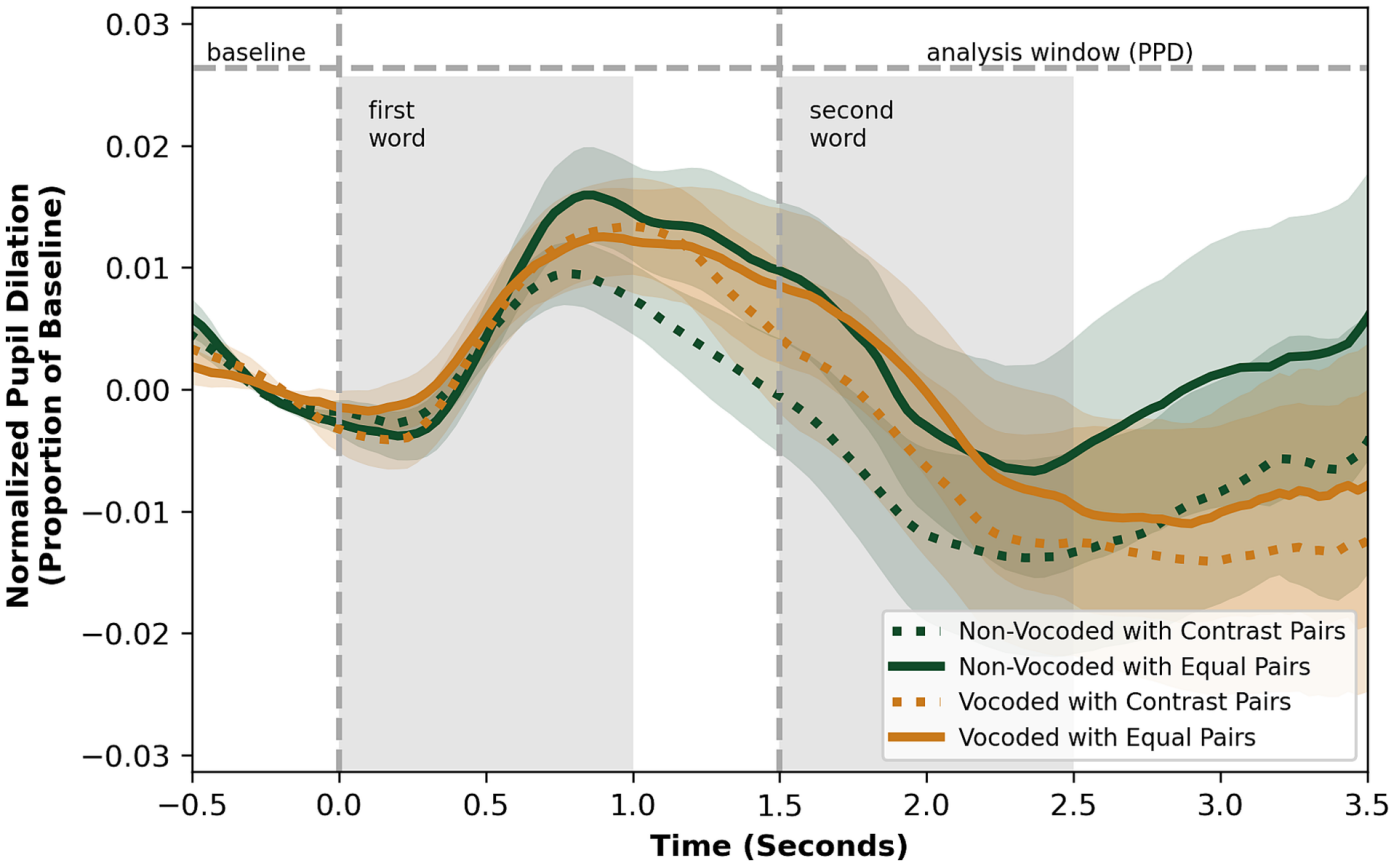
Pupil size over time in vocoded and non-vocoded conditions, for the phonological discrimination task, aggregated between participants.

**Figure 5:**
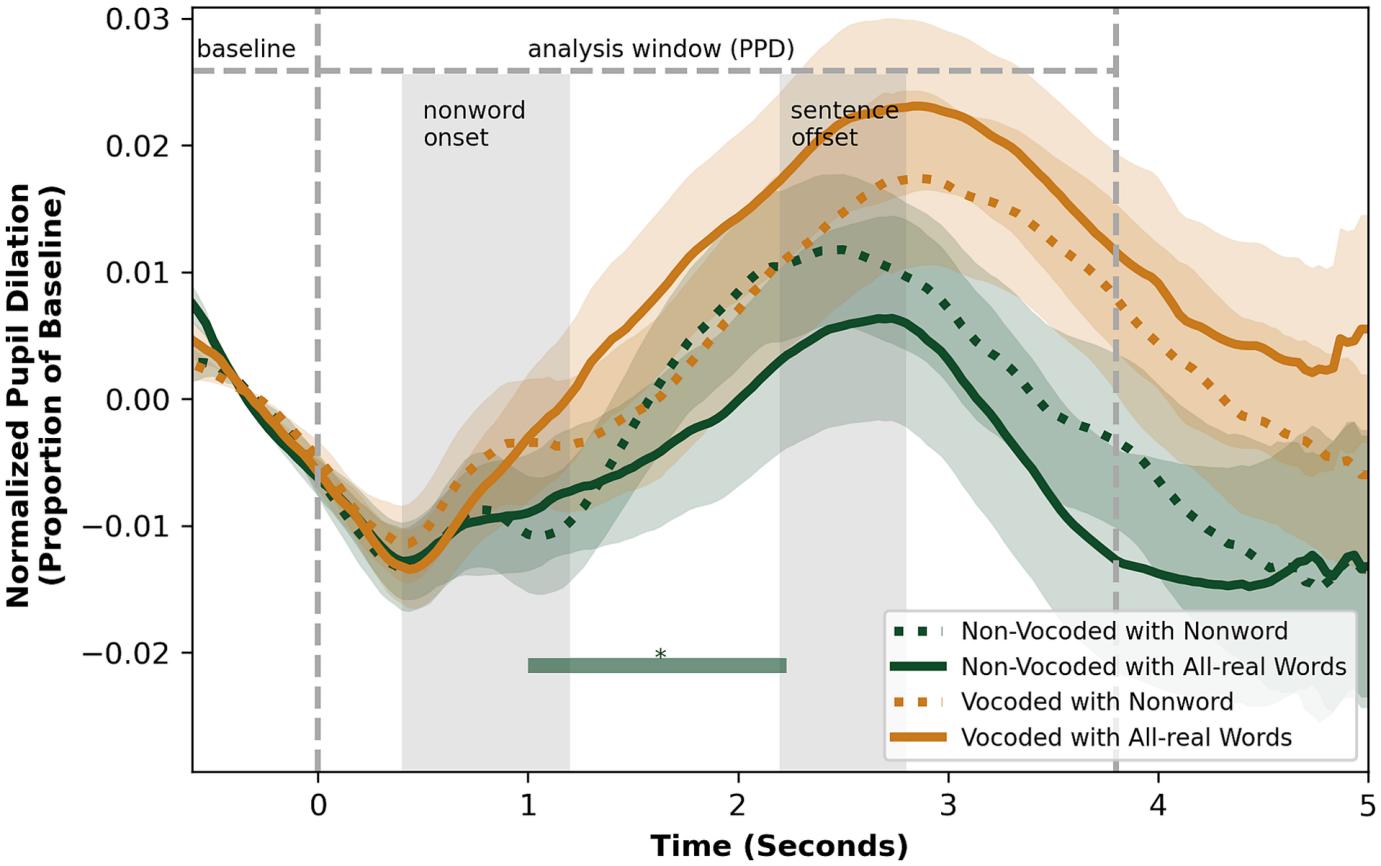
Pupil size over time in vocoded and non-vocoded conditions, for the detection task, aggregated between participants. * Timeframes with significant difference between nonword and all-real word sentence types in non-vocoded condition (*p* < 0.05).

The analysis of the pupil data was restricted to trials with correct responses to determine whether successful responses could be differentiated based on pupil dynamics. The aggregated pupillometry response traces across time for both tasks, encompassing data from all participants, are presented in Figure 4 and Figure 5 with respective detailed information in Table 1 and Table 2. In the phonological discrimination task, PPL occurred at a mean of 678 ms (SD = 867) after the presentation of the second word. In the detection task, the PPL for all conditions occurred at a mean of 2.04 s (SD = 1.07) after the onset of the nonword, or after 2.18 s (SD = 1.13) of the onset of the second word for all-real-word sentences when the same alignment was used, which aligns roughly with the offset of the sentence.

**Table 1:** Pupillometry measures for the phonological discrimination task in the non-vocoded and vocoded conditions, for each pair type.

Condition		Contrast		Equal		*t* (18)/*z*	*p*	n
		M	SD	M	SD			
Non-vocoded	PPD (%)	0.13	0.34	0.24	0.41	-1.2^a^	0.247	19
	PPL (ms)	516	811	787	926	12.5^b^	0.037^*^	19
Vocoded	PPD (%)	0.22	0.46	0.17	0.34	1.06^a^	0.303	19
	PPL (ms)	592	844	817	919	14.5^b^	0.054	19

Statistical tests: ^a^ paired t test; ^b^ Wilcoxon signed ranks test; PPD = peak pupil dilation; PPL = peak pupil latency.

^*^p < 0.05.

**Table 2:** Pupillometry measures for the detection task in the non-vocoded and vocoded conditions, for each pair type.

Condition		Nonword		All-real		*t* (21)/*z*	*p*	n
		M	SD	M	SD			
Non-vocoded	PPD (%)	0.20	0.36	0.05	0.37	2.32^a^	0.030^*^	22
	PPL (ms)	1844	1,090	2013	1,190	96.0^b^	0.498	22
Vocoded	PPD (%)	0.25	0.39	0.28	0.42	−122.0^b^	0.898	22
	PPL (ms)	2,233	1,030	2,342	1,067	−0.31^a^	0.754	22

Statistical tests: ^a^ paired t test; ^b^ Wilcoxon signed ranks test; PPD = peak pupil dilation; PPL = peak pupil latency.

^*^p < 0.05.

#### 3.2.1. Differences in pupil responses due to phonological contrast

In the non-vocoded condition, pupil parameters were sensitive to the presence or absence of the phonological contrast. In the phonological discrimination task, the PPL for pairs without contrast occurred, on average, 217 ms later than pairs with contrast, while PPD values had no significant difference (Table 1). A logistic regression analysis showed no significant effects on the pupil curve intercept, slope, quadratic term, or their interactions, between pairs with and without contrast, *χ*2 (7, *n* = 44) = 42.6, *p* = 0.67. In the detection task, participants exhibited greater PPD for sentences containing a nonword compared to sentences containing all real words, with no significant difference in PPL (Table 2). The differences in pupil dilation were significant in the interval of 610 ms to 1750 ms after the nonword onset (Figure 5), although the logistic regression analysis did not show any significant differences in the parameters of the fitted curve between trials with and without the phonological contrast, *χ*2 (7, *n* = 37) = 33.9, *p* = 0.45. No differences between trials with or without the phonological contrast were found for the vocoded stimuli.

#### 3.2.2. Differences in pupil responses due to speech degradation

In contrast to the perceptual results, there was no effect of speech degradation on pupil measures in the phonological discrimination task. This was found when analyzed with a linear mixed effects model that included auditory condition (vocoded or non-vocoded) and pair type (phonological contrast present or absent) as fixed effects, and participant as a random effect (models’ marginal *R*^2^s = 0.001 and 0.021, conditional *R*^2^s = 0.394 and 0.311, for PPD and PPL, respectively).

For the detection task, a higher PPD (*β* = 0.014, *p* = 0.007) was seen as an effect of speech degradation, when considered in a similar condition x pair type linear mixed effects model, with participants as random effects (marginal *R*^2^ = 0.037, conditional *R*^2^ = 0.611). There was no effect of vocoding on PPL (*β* = 0.359, *p* = 0.102, marginal *R*^2^ = 0.032, conditional *R*^2^ = 0.103).

## 4. Discussion

This study aimed to investigate the sensitivity of pupil temporal dynamics to multiple levels of auditory processing of phonological information. At the low-level, by analyzing the possibility of detecting phonological discrimination in the absence of lexical context, and at the high-level by directing attention to the phonological contrasts in the presence of context and lack of complete acoustic information.

### 4.1. Performance results

Performance data showed similar performance in the perception of phonological contrasts in both isolated nonword-pairs and nonwords embedded in sentences. Performance was equally affected by speech degradation, as indicated by changes in accuracy and *d’* scores in the vocoded speech condition. Although performance was poorer when the stimuli were degraded with a vocoder, participants were still able to perform the task above-chance, with over 70% accuracy. These results demonstrate participants’ ability to utilize both low- and high-level strategies to perform speech tasks effectively. However, it indicates that accuracy and sensitivity alone do not provide sufficient information to differentiate between the type of strategy used by individual participants.

The false alarm rate in the vocoded phonological discrimination task (Figure 2) suggests that perceiving two speakers as producing the same nonword was challenging in the degraded speech condition, although the material used in this study has been evaluated as not containing ambiguous phonemes in unprocessed speech (Nielsen and Dau, 2019). False alarms occur when pairs were mistakenly perceived as having a contrast when they did not contain a contrast (i.e., two different speakers producing the same word), revealing a failure to perceive stable signal characteristics during phoneme recognition. Moreover, single-word recognition under vocoded condition appeared as a predictive factor for phonological discrimination, indicating similarity in the tasks’ underlying processes. Participants in both tasks were forced to rely on the variable acoustic characteristics of the speech signal to make phonological decisions, and failures occurred unbiased, regardless of the presence or absence of contrast.

Previous studies of phoneme confusion, employing similar 8-channel noise vocoders, have documented higher consonant recognition performance compared to the results observed in this study (Friesen et al., 2001; Xu et al., 2005; Zhou et al., 2010; Jahn et al., 2019; Goupell et al., 2020). These prior studies reported consonant recognition accuracy ranging from approximately 60% (Zhou et al., 2010) in ‘consonant-vowel’ contexts, to 68% for monosyllables (Goupell et al., 2020) and around 80% (Xu et al., 2005; Jahn et al., 2019) within ‘vowel-consonant-vowel’ contexts. However, those studies have used a closed set of syllables or words for the consonant recognition task. The open-set word recognition used in our study was more difficult for participants when attempting to identify the target word. The open-set task increased the number of potential responses, which enhances the activation of neighboring words in the word recognition task. Moreover, for the discrimination of contrasts, the vocoder might be more detrimental to the identification of initial consonants rather than medial consonants, as the highest accuracies were reported in studies using medial consonant identification (Friesen et al., 2001; Jahn et al., 2019). In medial positions, the transition information from vowel to consonant is readily available and contributes to phoneme recognition (Xu et al., 2005). Therefore, the participants’ ability to predict the consonants may have been compromised in our study, shown by the reduced accuracy scores in the phonological discrimination task.

As expected, the presence of context led to a bias toward reporting nonwords as real words in the vocoded detection task, causing the participants to ignore or miss the phonological contrast. A degraded signal amplifies the perceptual bias in phonological perception, increasing the reliance on non-acoustic information such as lexical information and context when categorizing phonological contrasts (Gianakas and Winn, 2019; Vickery et al., 2022; Winn and Teece, 2022), producing the effects observed.

### 4.2. Pupillometry responses

#### 4.2.1. Differences in pupil responses due to phonological contrast

The presence of a phonological contrast did not elicit higher pupil dilation in the phonological discrimination task, as it would be expected in a presence of a variant stimuli (Wagner et al., 2016; Kinzuka et al., 2020). The differences here can be attributed to the demands of the tasks. The simplicity of the forced-choice task might not have elicited sufficient differences in the demand for cognitive processing to capture the effect of the phonological contrast. Additionally, in contrast to previous studies which used words as material for discrimination, in our study the participants could not use lexical information to support the decision regarding the change in the phoneme category. Therefore, their judgment was forced to occur solely at the phonological level. The absence of a significant difference in pupil parameters suggests that pupil dynamics may be more sensitive to higher-level cognitive and language processing, as to lexical categorization (Kamp and Donchin, 2015), rather than lower-level phonological categorization.

Moreover, the pupil response to phonological contrasts may be indistinguishable from the response for the perception of acoustic contrasts. The contrast between two speakers in our paradigm, one in each token in the nonword-pair, was done to ensure that discrimination was occurring at a phonological rather than acoustical level. It is known that different speakers possess a natural variability in multiple acoustic domains as voice-onset-time, vowel formants, consonant intensity, among others (Allen et al., 2003; Christiansen and Henrichsen, 2011). Therefore, identifying two nonwords as the same would require their processing at the phonological level. However, as the pupil dilates for acoustic deviants, such as pure tones and noise varying in frequency (Liao et al., 2016; Selezneva et al., 2021), pupil dilation could also be an index of the processing of the dynamic acoustic characteristics of speech in an effort to solve ambiguity caused by interspeaker variations in phoneme production and boundaries (Lewis and Bidelman, 2020; Winn, 2020; Reese and Reinisch, 2022; Yu, 2022). Such a response to acoustic differences would explain the comparable PPDs recorded for both pairs with and without phonological contrast, since for both types of pairs the acoustic variability was present.

Interestingly, in the phonological discrimination task, PPLs were shorter for pairs with phonological contrast than for pairs without contrast. Koelewijn et al. (2017) describe the PPL as a measure of the speed of cognitive processing, with shorter latencies indicating faster cognitive processing or the need for processing less information. One explanation for our results is that to correctly identify a phonological contrast, the participant would only need to identify the first phoneme of the second word in the pair, but to correctly identify the absence of a contrast required the processing of the whole nonword in a pair. Therefore, a decision could be taken quicker with far less information for pairs with contrast.

The presence of context, in the detection task, led to higher PPD in sentences containing a phonological altered word (nonword). This effect was expected as it had been previously reported by Wagner et al. (2016) and Winn and Teece (2021, 2022). These studies found that substituted and distorted phonemes within words in a sentence lead to steeper pupil dilation. As discussed in Winn and Teece (2022), the presence of sentence context makes it difficult to determine if the higher dilation occurs due to increased cognitive demand for sentence processing introduced by the ambiguous lexical entry, or due to the detection of the phonological contrast. However, the absence of difference in the results of the phonological discrimination task suggests that the pupil response may be more closely linked to the violation of the lexical expectation rather than the phonological contrast.

Remarkably, in our study, participants were not asked to process the whole sentence in any manner (they did not repeat it back, nor derived its meaning). Therefore, it could be expected that after detecting the nonword in the second position of the sentence, the participants’ demand for processing would immediately decrease, which should have resulted in a reduction in the pupil size. Yet, the observed pupil behavior indicates that the whole sentence was processed before the response was given. Despite the different protocols used, these results are consistent with Winn and Teece (2021, 2022), in which participants were asked to repeat the whole sentence back to the experimenter. These findings suggest that, despite being instructed to track individual words in the sentence, listeners may have used the whole sentence context to make decisions regarding the presence or absence of the phonological contrast. As an anecdotal report, during the experiment session, several participants reported attempting to ‘repair’ the nonword or ‘figure out the correct word’.

#### 4.2.2. Differences in pupil responses due to speech degradation

The results in the vocoded condition support the argument that the pupil response reflects processing at the lexical and sentence level. The high accuracy scores for identifying the presence of a nonword within a sentence shows that participants were able to detect the phonological alteration despite the vocoded speech, indicating that phonological discrimination was occurring at a low-level. However, the lack of difference in the pupil parameters between sentences with and without phonologically modified words suggests that the pupil response captured the increase in cognitive processing required to understand the vocoded sentences, rather than the detection of a phonological contrast. Furthermore, the trend of interpreting nonwords as real words in the performance results suggests that the participants were likely attempting phonological restoration throughout the vocoded experiment. In other words, it is possible that the absence of differences in the pupillary response between stimuli with and without phonological contrast reflects the registration of a different type of response besides the detection of the contrast. The physiological mechanisms underlying pupil dilation are also involved in the process of decision-making (Kafkas and Montaldi, 2018). As such, when decisions require greater cognitive processing and memory demand, pupil size increases. It is important to note that the signal restoration of the vocoded stimuli comes at a cost even for real words (Winn et al., 2015; Balling et al., 2017). This global response, which is related to the processing of the auditory stimulus as a whole, may be more pronounced than the response to the detection of the phonological contrast, thereby masking its signal.

Another possible explanation for the lack of difference in pupil metrics between stimuli with or without phonological contrast is that errors in detecting the contrast may have occurred at different moments in the stimuli presentation. Since participants were not instructed about the possible location of the phonological contrast, it was not possible to track the exact moment when errors occurred. As a result, the effect of the phonological contrast may have been distributed across the time series average (Winn and Teece, 2021), which could not be tracked by our analysis.

### 4.3. Study limitations

As in any forced-choice task, the methodology used opens the possibility for participants to ‘guess’ the responses. This effect can be considered during the signal detection analysis of the performance but might influence the amplitude and morphology of the pupil responses. Responses based on chance, with low or no processing of the stimulus, can contaminate the time-series average during pupil analysis and effects be missed. Additionally, pupil responses are modulated by the sympathetic nervous system, which can be influenced by a range of factors such as engagement, fatigue, or self-perception of performance (Hopstaken et al., 2015; Zekveld et al., 2018; McGarrigle et al., 2021). Although we attempted to counterbalance for fatigue effects by randomizing the order of the presentation of the tasks and stimuli, it is possible that the low scores in speech perception, achieved in the vocoded speech condition, have led to disengagement from the task, which would be reflected in an overall reduction of pupil dilation (Hopstaken et al., 2015; Ohlenforst et al., 2017).

It is worth noting that the characteristics of the phonological contrast in the phonological discrimination task and the detection task were not the same. While in the phonological discrimination task the contrast was defined by a change in one production feature, multiple production features were modified in the detection task. In terms of acoustic differences, this might mean that the acoustic degradation would affect different aspects of the phonological perception in each task (Xu et al., 2005; Zhou et al., 2010). Furthermore, it raises the possibility that pupil dilation would be sensitive to the distance between the expected stimulus and the contrast, as previously observed for non-speech stimuli (Liao et al., 2016; Winn and Teece, 2021).

Furthermore, participants in our study were exposed to only a brief practice session with the vocoded stimuli. While this training was conducted similarly as previous studies (Hervais-Adelman et al., 2011; Winn et al., 2015), adapting to vocoded speech may require longer practice (Hervais-Adelman et al., 2011). Thus, it is possible that the immediate results produced by the spectral degradation would not have been sustained in a longer task, which would have induced phonological accommodation and potentially have led to better speech recognition (Jesse, 2021).

## 5. Conclusion

The present study offers insights on the pupil temporal dynamics from the processing of phonological information. The lack of differences in the pupil dilation to the presence of a phonological contrast in lexically decontextualized nonwords (phonological discrimination task) could suggest that pupil dynamics are more sensitive to higher-level cognitive and language processing, such as lexical categorization, rather than lower-level phonological categorization. Nevertheless, the pupil response to phonological contrasts may overlap with responses to acoustic differences, indicating that pupil dilation may reflect the processing of dynamic acoustic characteristics of speech.

In the presence of lexical/contextual information (detection task), phonological contrasts led to higher pupil dilation. This increase in pupil dilation could be attributed either to an increase in cognitive demand for processing a sentence containing a nonword, or to a response to the detection of the phonological contrast. The inability to distinguish between high and low-level processing in the detection task stemmed from participants’ apparent reliance on sentence context when making decisions about phonological contrasts, despite explicit instructions to track individual words.

These findings bring important considerations to the use of pupillometry when investigating phonological perception in the presence of lexical meaning or acoustic variability. Further research is needed to gain a comprehensive understanding of the intricate interactions among acoustic, phonological, and linguistic factors and their influence on pupil dynamics during speech perception.

## Data availability statement

The raw data supporting the conclusions of this article will be made available by the authors, without undue reservation.

## Ethics statement

The requirement of ethical approval was waived by the Scientific Ethics Committees, Center for Regional Development, Capital Region - Denmark for the studies involving humans because the Scientific Ethics Committees, Center for Regional Development, Capital Region - Denmark considered it to be a study in the social domain. The studies were conducted in accordance with the local legislation and institutional requirements. The participants provided their written informed consent to participate in this study.

## Author contributions

JC, FP, EN, KF, and BL: conceptualization and design. JC: data collection and statistical analysis and writing—original draft preparation. FP, EN, KF, and BL: writing—review and editing. All authors approved the submitted version.
